# NANOG Plays a Hierarchical Role in the Transcription Network Regulating the Pluripotency and Plasticity of Adipose Tissue-Derived Stem Cells

**DOI:** 10.3390/ijms18061107

**Published:** 2017-05-23

**Authors:** Maria Pitrone, Giuseppe Pizzolanti, Laura Tomasello, Antonina Coppola, Lorenzo Morini, Gianni Pantuso, Romina Ficarella, Valentina Guarnotta, Sebastio Perrini, Francesco Giorgino, Carla Giordano

**Affiliations:** 1 Aldo Galluzzo Laboratory of Regenerative Medicine, Section of Endocrinology, Diabetology and Metabolism, Di.Bi.M.I.S, University of Palermo, 90127 Palermo, Italy; maria.pitrone@unipa.it (M.P.); laura.tomasello@unipa.it (L.T.); antonina.coppola02@unipa.it (A.C.); valentina.guarnotta@unipa.it (V.G.); 2 Oncology Surgery, University of Palermo, 90127 Palermo, Italy; loremor86@yahoo.it (L.M.); gianni.pantuso@unipa.it (G.P.); 3 Endocrinology and Metabolic Diseases, University of Bari, 70124 Bari, Italy; r.ficarella@endo.uniba.it (R.F.); sebastio.perrini@uniba.it (S.P.); francesco.giorgino@uniba.it (F.G.); 4 ATeN (Advanced Technologies Network Center), University of Palermo, 90127 Palermo, Italy

**Keywords:** adipose derived stem cell (ASC), regenerative medicine, embryonic stem cell marker network

## Abstract

The stromal vascular cell fraction (SVF) of visceral and subcutaneous adipose tissue (VAT and SAT) has increasingly come into focus in stem cell research, since these compartments represent a rich source of multipotent adipose-derived stem cells (ASCs). ASCs exhibit a self-renewal potential and differentiation capacity. Our aim was to study the different expression of the embryonic stem cell markers *NANOG* (homeobox protein NANOG), *SOX2* (SRY (sex determining region Y)-box 2) and *OCT4* (octamer-binding transcription factor 4) and to evaluate if there exists a hierarchal role in this network in ASCs derived from both SAT and VAT. ASCs were isolated from SAT and VAT biopsies of 72 consenting patients (23 men, 47 women; age 45 ± 10; BMI between 25 ± 5 and 30 ± 5 range) undergoing elective open-abdominal surgery. Sphere-forming capability was evaluated by plating cells in low adhesion plastic. Stem cell markers CD90, CD105, CD29, CD31, CD45 and CD146 were analyzed by flow cytometry, and the stem cell transcription factors NANOG, SOX2 and OCT4 were detected by immunoblotting and real-time PCR. *NANOG*, *SOX2* and *OCT4* interplay was explored by gene silencing. ASCs from VAT and SAT confirmed their mesenchymal stem cell (MSC) phenotype expressing the specific MSC markers CD90, CD105, NANOG, SOX2 and OCT4. *NANOG* silencing induced a significant *OCT4* (70 ± 0.05%) and *SOX2* (75 ± 0.03%) downregulation, whereas *SOX2* silencing did not affect *NANOG* gene expression. Adipose tissue is an important source of MSC, and siRNA experiments endorse a hierarchical role of *NANOG* in the complex transcription network that regulates pluripotency.

## 1. Introduction

Both visceral (VAT) and subcutaneous adipose tissues (SAT) represent an alternative source of mesenchymal stem cells (MSCs). Adipose-derived stem cells (ASCs) have been proposed by the International Fat Applied Technology Society as a plastic-adherent, proliferative, multipotent cell population isolated from adipose tissue [1,2]. ASCs have a fibroblastic-like morphology and possess the properties of MSCs traditionally isolated from bone marrow [3,4]. Furthermore, ASCs have a remarkable potential to differentiate in vitro towards the osteogenic, adipogenic, myogenic and chondrogenic lineages when maintained under specific culture conditions [5]. Nowadays, there is great interest in understanding more in detail the cellular and molecular mechanisms of ASCs, which are known to modulate self-renewal and differentiation properties. Indeed, the latter characteristics have made it possible to consider them as the preferable candidate for employment in regenerative medicine [6]. The role of the transcription factors that regulate self-renewal and differentiation is well known in embryonic stem cells [7]. Boyer et al. showed that *OCT4* (octamer-binding transcription factor 4), *NANOG* (homeobox protein NANOG) and *SOX2* (SRY (sex determining region Y)-box 2) work as a trio to support each other’s expression and that of other self-renewal genes repressing differentiation genes [8], while other studies suggested that only *OCT4* and *NANOG* are co-regulated in embryonic stem cells [7]. However, until today, the exact role of *NANOG*, *OCT4* and *SOX2* in adult stem cells isolated from different tissues has not been well identified and is still controversial [9,10]. For example, knockout of *OCT4* did not affect their capacity for colony formation and differentiation into bone, fat and cartilage [11]. In addition, many studies showed the existence of pseudogenes for *OCT4* [12] and *NANOG* that are not expressed in the nucleus, but in cytosol [9,13], whose function has not so far been fully explained. Hyslop et al. suggested that *NANOG* acts as a gatekeeper of pluripotency in human embryonic development [14]. In this case, downregulation of *NANOG* in human embryonic stem cells (ESCs) induces upregulation of endoderm- and trophectoderm-associated genes [13,14,15]. As regards hASCs, the single components of the trio have been described separately, but the possible interplay among the principal transcription factors has not been fully explored.

Our aim was to study the expression of *NANOG*, *SOX2* and *OCT3/4* in ASCs derived from SAT and VAT and to evaluate whether there exists a transcription factor with a more prominent role in this network.

## 2. Results

### 2.1. Isolation of Adipose Derived Stem Cells

**Figure 1:**
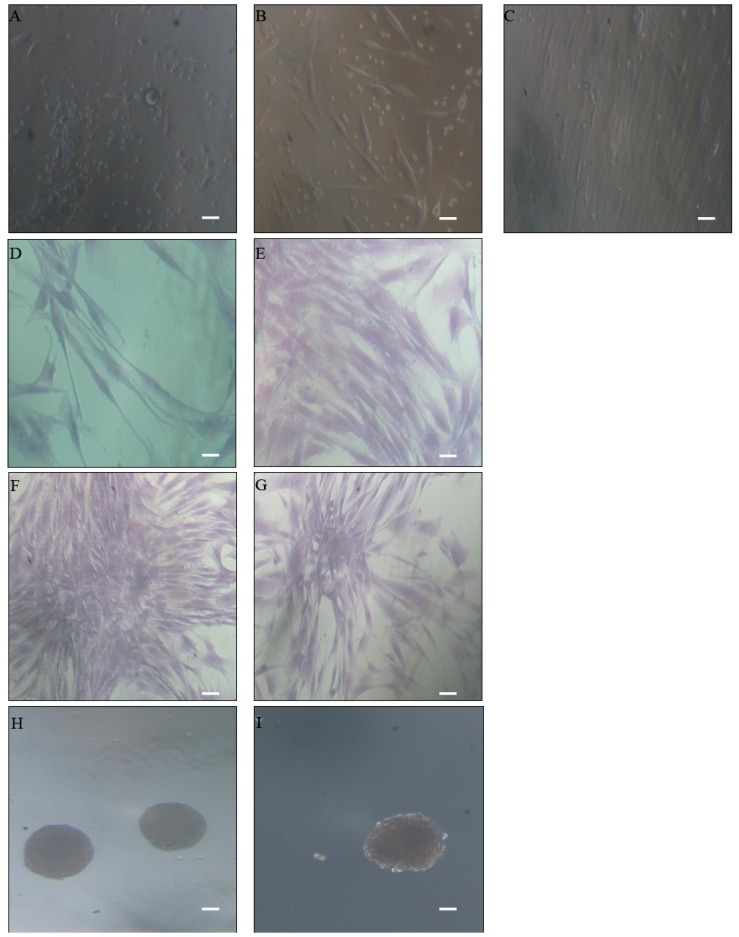
Morphology of adipose-derived stem cells (ASCs) under light microscopy (10×) with phase contrast with a Nikon DS-FI1 CCD camera. Scale bars: 400 µm. Stromal vascular cell fraction (SVF) morphological characteristics on day 2 (**A**), day 5 (**B**) and day 10 (**C**) of expansion cultures. Crystal violet staining shows visceral adipose tissue mesenchymal stem cells (V-ASCs) having large nuclei and extended cytoplasms (**D**) in comparison to subcutaneous adipose tissue mesenchymal stem cells (S-ASCs), which have large nuclei and globular cytoplasms (**E**); (**F**,**G**) show similar aggregation of MSCs from V-ASC and S-ASC in adhesion cultures; (**H**,**I**) show spheres from V-ASCs and S-ASCs in low adhesion cultures. An experiment representative of 72 samples studied is shown.

Enzymatic digestion of biopsied human adipose tissue was obtained from 72 consenting patients (23 men and 47 women; age 45 ± 10 years; with body mass index (BMI) range 25 ± 0.5 and 35 ± 5, respectively) undergoing elective open-abdominal surgery. The freshly-isolated stromal vascular cell fraction (SVF) was a compounded cell population with a spindle, triangular, polygonal or round shape, which contained fibroblasts and adipocytes at different differentiation stages. These cells adhered to a flask without a substrate. After 48 h, observation under a light microscope showed a round and small morphology (Figure 1A), whilst after five days (Figure 1B), these cells extended and showed a spindle shape. After seven days, the number of some mature cells, such as mature adipocytes, decreased, and the morphology of most cells tended to be uniform. After 10 days (Figure 1C), colonies of fibroblastic-like cells were observed, although fibroblasts mixed in ASCs were still found. Both of these cell types reached confluence after about 15 days. MSCs derived from visceral adipose tissue (V-ASCs) were characterized by large nuclei and elongated cytoplasms (Figure 1D) and those derived from subcutaneous adipose tissue (S-ASCs) by large nuclei and globular cytoplasms (Figure 1E). Both S-ASCs and V-ASCs formed cell aggregation at the third passage (Figure 1F,G) and in low-adhesion culture conditions formed spheres, suggesting the stem origin of the cells (Figure 1H,I).

### 2.2. Cell Cycle Analysis

**Figure 2:**
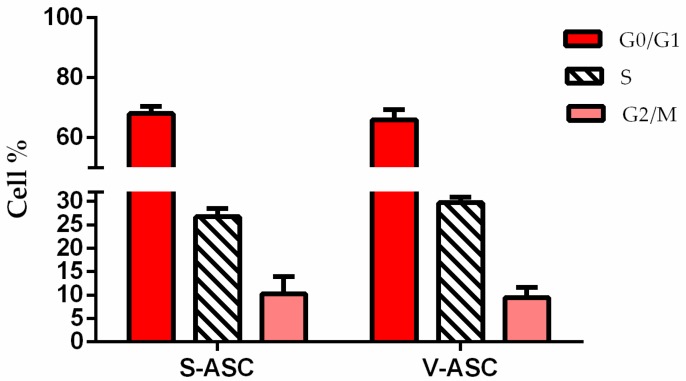
Cell cycle distribution of S-ASCs and V-ASCs. Both cell types were analyzed according to Nicoletti’s protocol [16]. Twenty-five samples of S-ASCs and V-ASCs cultured in adhesion condition at passage 3 were analyzed.

Cell cycle analysis at passage 3 showed no significant different distribution of cells in the G1, G2 and S phases in S-ASC and V-ASC. S-ASC in the G1 phase was 68.10 ± 2.32% and V-ASC was 66.01 ± 3.43%. The proliferation index (PI), expressed as % G2 + % M, was 10.3 ± 3.7% and 9.46 ± 2.14%, respectively, for S-ASC and V-ASC (Figure 2).

### 2.3. Flow Cytometry Stem Cell Phenotype Characterization

**Figure 3:**
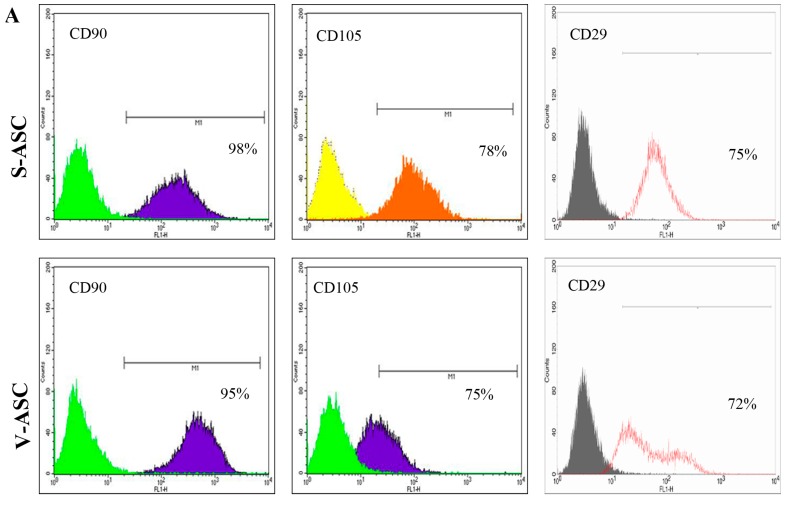

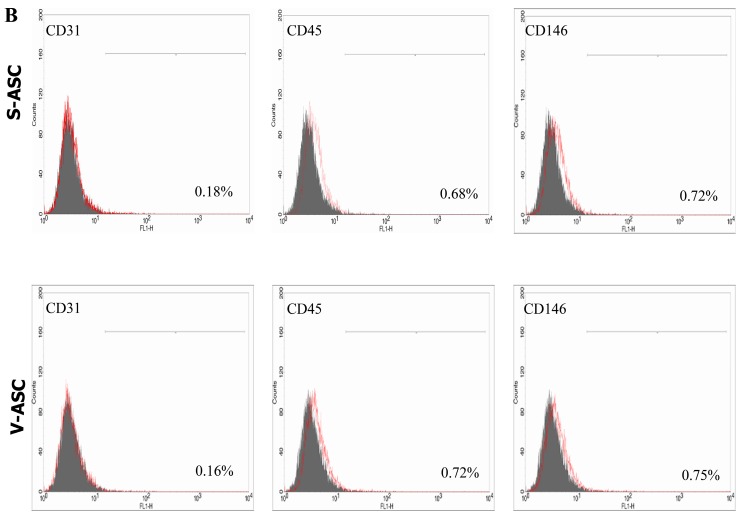
(**A**) Cytofluorimetric analysis for CD90, CD105 and CD29; (**B**) The cells are negative for CD31, CD45 and CD146 in S-ASC and V-ASC. All fields are representative of one S-ASC and V-ASC sample out of at least 12 independent experiments.

The cell-surface antigenic characteristics of 12 samples of S-ASCs and 12 samples of V-ASCs at passage 3 were analyzed by flow cytometry. Both populations were positive for CD90 (94 ± 3.9% and 91 ± 3.6%, respectively), CD105 (76 ± 4.2% and 73 ± 2.7%, respectively) and CD29 (72 ± 2.5% and 70.8 ± 2.8%, respectively) (Figure 3A) and showed almost no expression of CD31, CD45 and CD146 (<1%) (Figure 3B).

### 2.4. Stem Cell Markers of S-ASC and V-ASC Grown as Adherent Cells or Spheres

**Figure 4:**
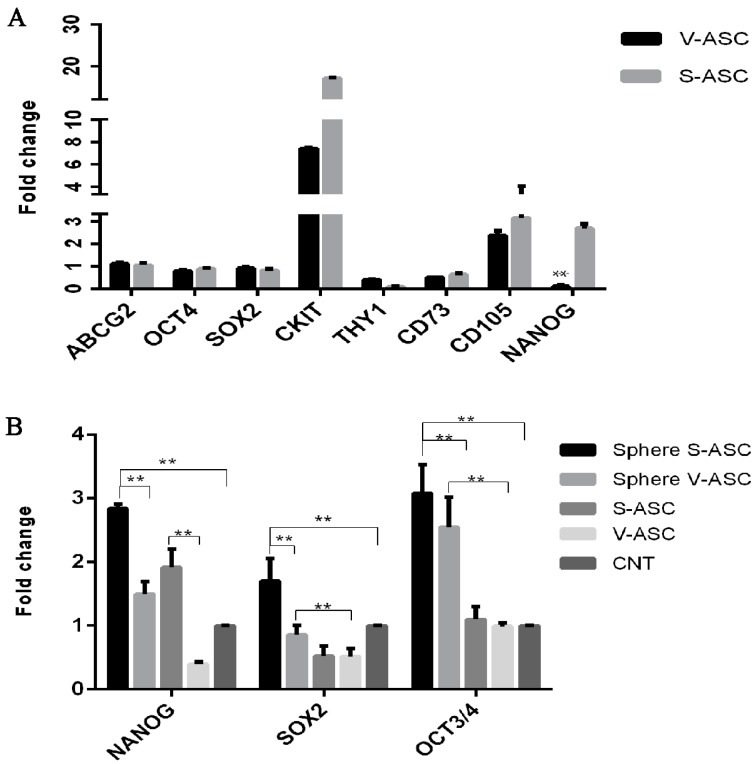
(**A**) qRT-PCR analyses in S-ASC and V-ASC cells cultured in adhesion condition. Data are representative of three independent experiments. Relative expression levels for *ABCG2*, *OCT4*, *SOX2*, *CKIT*, *THY1*, *CD73*, *CD105* and *NANOG* were assessed using the 2^−ΔΔ*C*t^ method. Values shown as mean ± SE, ** *p* < 0.01. The data shown are relative to an endogenous control (beta-actin), with the fold change compared to expression levels in commercial bone marrow-mesenchymal stem cells (set to one); (**B**) qRT-PCR analysis in spheres from S-ASC and V-ASC primary cells. Data are representative of three independent experiments with the fold change compared to expression levels in commercial human adipose derived stem cell (ASC52telo, hTERT immortalized adipose-derived mesenchymal stem cells) (set to one). Values shown as the mean ± SE, ** *p* < 0.01. CNT, control.

To assess the multipotent state of ASCs from subcutaneous and visceral adipose tissue, we detected some of the embryonic stem cell markers, such as *ABCG2*, *OCT4*, *SOX2*, *C-KIT*, *THY1*, *CD-73*, *CD-105* and *NANOG*, using mRNA samples from 10 S-ASCs and 10 V-ASCs cultured in adhesion conditions. Expression analysis (see Table S1 in supplementary) showed only a significant difference in *NANOG* mRNA levels in S-ASC vs. V-ASC. ASCs from VAT and SAT expressed all of the examined stem cell markers (Figure 4A).

Furthermore, we compared the stem cell transcription factor expression pattern in spheres derived from S-ASCs and V-ASCs vs. S-ASCs and V-ASCs in adhesion condition cultures. We found that *NANOG*, *SOX2* and *OCT4* were expressed at higher levels in spheres from S-ASC and V-ASC cells in comparison to the same cells in adhesion conditions (see Table S2 in supplementary). In addition, spheres from S-ASCs were characterized by the highest values, as shown in Figure 4B (*p* < 0.01).

**Figure 5:**
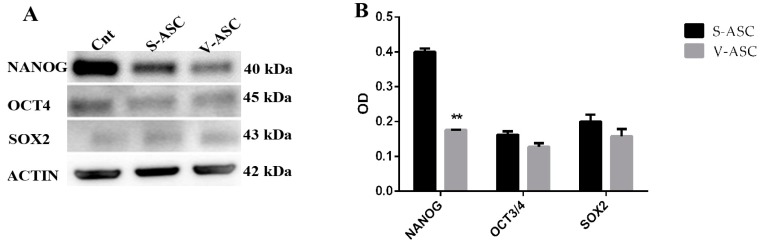
(**A**) Representative Western blot analysis in the S-ASC and V-ASC cells of NANOG, OCT3/4 and SOX2; (**B**) densitometric analysis of the Western blot depicted in (**A**). The histograms are the results of three independent experiments. The values are shown as the mean ± SE, ** *p* < 0.01. OD, optical density (** *p* < 0.01).

Western blot analysis confirmed that NANOG was more expressed in ASCs isolated from SAT than from VAT (*p* < 0.01) (see Table S3 in supplementary) (Figure 5A,B).

### 2.5. NANOG and SOX2 Silencing in S-ASC and V-ASC

**Figure 6:**
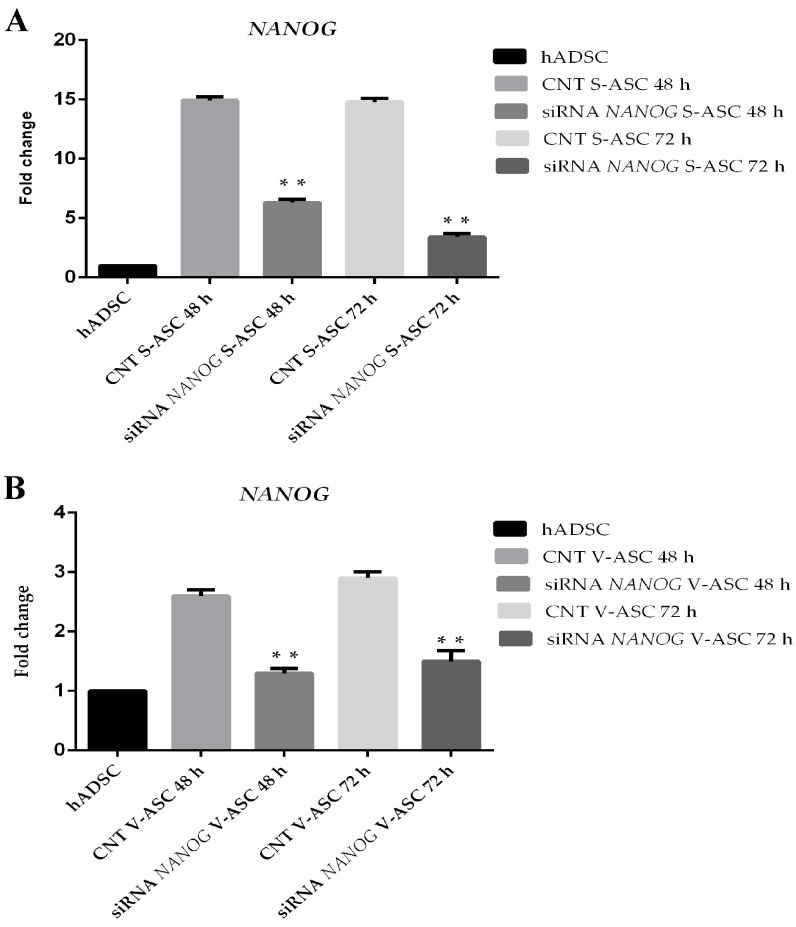
(**A**) *NANOG* silencing in S-ASCs after 48 and 72 h; (**B**) *NANOG* silencing in V-ASCs after 48 and 72 h. Fold change was calculated using the 2^−ΔΔ*C*t^ method. Data representative of three independent experiments with the fold change compared to expression levels in a commercial human adipose-derived stem cell line (ASC52telo, hTERT immortalized adipose-derived mesenchymal stem cells). Values shown as the mean ± SE, ** *p* < 0.01.

**Figure 7:**
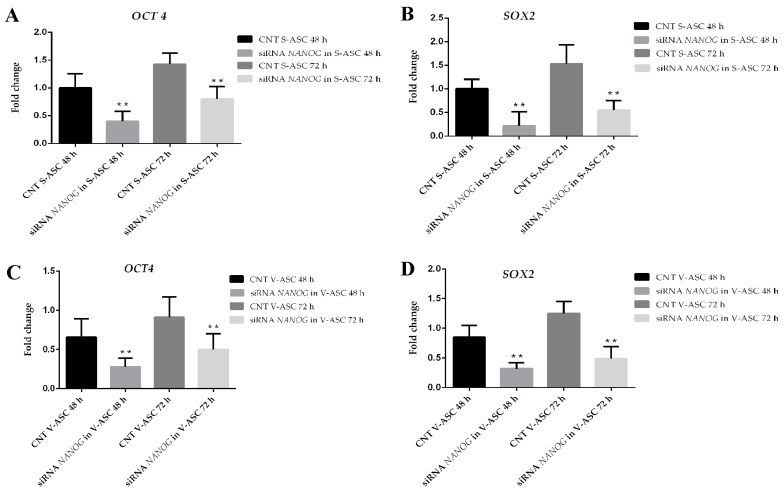
(**A**) qRT-PCR analysis in S-ASCs of the *OCT4* gene after *NANOG* silencing; (**B**) qRT-PCR analysis in S-ASCs of the *SOX2* gene after *NANOG* silencing; (**C**) qRT-PCR analysis in V-ASCs of the *OCT4* gene after *NANOG* silencing; (**D**) qRT-PCR analysis in V-ASCs of the *SOX2* gene after *NANOG* silencing. *NANOG* silencing was assessed with stealth siRNA (siRNANANOG) vs. siCONTROL treated cells (CNT). The data are representative of three independent experiments. The values are shown as the mean ± SE, ** *p* < 0.01.

To establish the relationship between *SOX2*, *OCT-4*, *NANOG*, we evaluated their expression after *NANOG* silencing. *NANOG* silencing (Figure 6A,B) in S-ASCs after 48 h (73 ± 0.03%) and after 72 h (70 ± 1.23%) and in V-ASCs cultured in adhesion after 48 (70 ± 1.4%) h and 72 h (72 ± 2.3%) caused downregulation of *OCT3/4* (70 ± 0.05%, *p* < 0.01) and *SOX2* genes (75 ± 0.03%, *p* < 0.01) in S-ASCs (Figure 7A,B) and in V-ASCs (Figure 7C,D).

**Figure 8:**
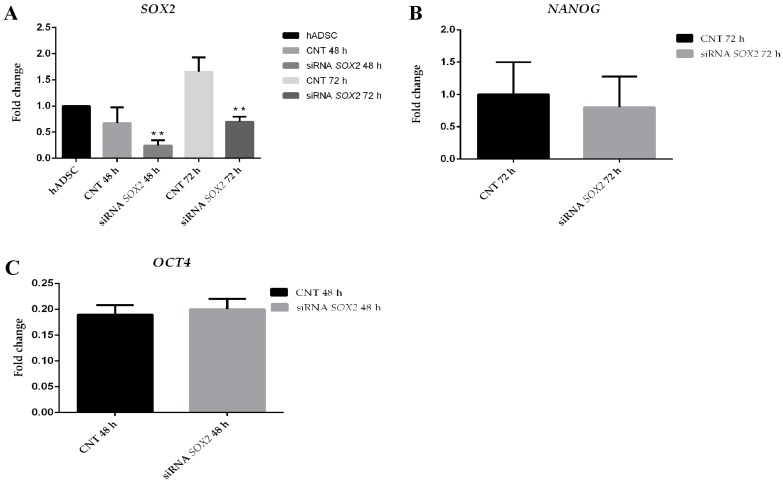
(**A**) *SOX2* silencing assessed using the 2^−ΔΔ*C*t^ method; (**B**) qRT-PCR analysis in S-ASCs of *NANOG* and *OCT4*; (**C**) gene expression after *SOX2* silencing with stealth siRNA (siRNASOX2) vs. siCONTROL treated cells (CNT). The data are representative of three independent experiments.

By contrast, *SOX2* silencing after 48 h (72 ± 1.5%) and 72 h (70 ± 2.3%) (Figure 8A) in S-ASCs cultured in adhesion showed no effect on *NANOG* and *OCT4* expression after 48 h (Figure 8B,C).

**Figure 9:**
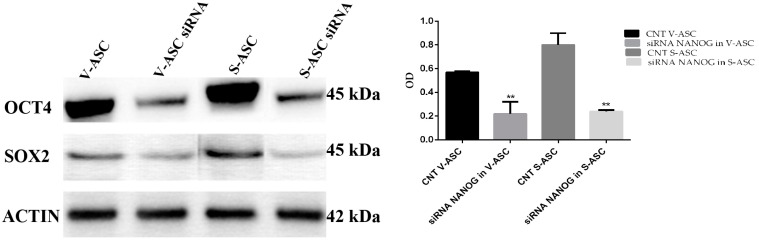
Western blot of OCT4 and SOX2 with proteins extracted from V-ASC and S-ASC cells after NANOG silencing with stealth siRNA vs. siCONTROL treated cells.

Western blot analysis of SOX2 and OCT3/4 after NANOG silencing with stealth siRNA after 96 h in S-ASCs (optical density (OD) = 0.8 ± 0.1 and 0.24 ± 0.01, *p* < 0.01) and in V-ASCs (OD = 0.5 ± 0.01 and 0.22 ± 0.01) caused downregulation of the OCT4 protein in S-ASCs (OD = 1.16 ± 0.17 vs. 0.38 ± 0.12; *p* < 0.01) and in V-ASCs (OD =1.09 ± 0.16 vs. 0.39 ± 0.12, *p* < 0.01). At the same time, downregulation of the SOX2 protein was observed in S-ASCs (OD: 0.49 ± 0.05 vs. 0.20 ± 0.02; *p* < 0.01) and in V-ASCs (OD: 0.5 ± 0.01 vs. 0.01 ± 0.001; *p* < 0.01) (Figure 9).

**Figure 10:**
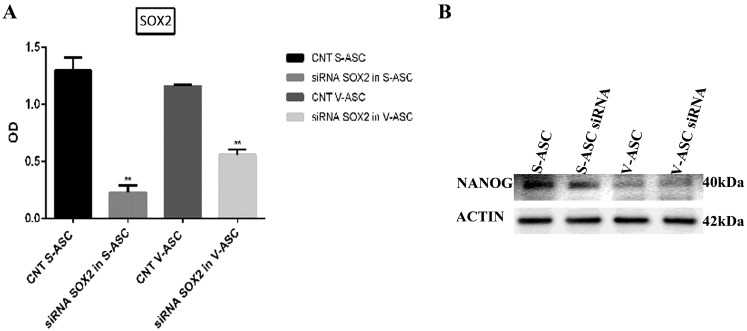
(**A**) Analysis of SOX2 silencing after 96 h; (**B**) Western blot of NANOG with proteins extracted from S-ASC and V-ASC cells after SOX2 silencing with stealth siRNA vs. siCONTROL (CNT) treated cells. The results were normalized with beta actin. The data are representative of three independent experiments.

By contrast, SOX2 silencing in S-ASC (OD = 1.3 ± 0.1 vs. 0.23 ± 0.06, *p* < 0.01) and in V-ASC cells (OD = 1.16 ± 0.013 vs. 0.56 ± 0.04; *p* < 0.01) (Figure 10A) after 96 h was not able to affect NANOG protein levels in S-ASC (OD = 1.17 ± 0.1 vs. 1.2 ± 0.01, *p* = NS) and in V-ASC (OD = 0.7 ± 0.2 vs. 0.68 ± 0.1; *p* = NS) (Figure 10B).

## 3. Discussion

Adipose tissue represents an interesting source of multipotent stem cells [5,16] and is considered fundamental for comprehension of adipose tissue biology under normal physiology. In addition, interest in disease state conditions is also developing, even though limited information is available on visceral and subcutaneous ASCs and the relationship between in vitro and in vivo adipogenesis.

The purpose of this work was to study the different expression of the embryonic stem cell markers *NANOG*, *SOX2* and *OCT3/4* and the role of NANOG in this network, evaluated in both S-ASCs and V-ASCs isolated from SAT and VAT in a large series of obese and non-obese subjects, who underwent elective open-abdominal and laparoscopy surgery. When isolated MSCs were cultured without any substrate [17], we observed morphological differences in V-ASC and S-ASC cells, which included cell shape and cell size in accordance with previous observations [18,19]. ASCs derived from subcutaneous adipose tissue were characterized by large nuclei and cytoplasms and formed cell aggregation (fusiform shape), whilst ASCs derived from visceral adipose tissue showed large nuclei and extended cytoplasms (classical fibroblast-like). Our data confirm that cells are capable of forming sphere clusters in a serum-free medium supplemented with fibroblast growth factor-basic (FGF-b) and epidermal growth factor (EGF) [20], and these spheres derived from S-ASCs and V-ASCs expressed all of the ESC markers evaluated, *SOX-2*, *OCT4* and *NANOG*, more markedly than S-ASCs and V-ASCs in adhesion, demonstrating that the spheres mostly possess stemness potential. Interestingly, during the time of floating culture, the spheres maintained the expression of stem cell markers. From our findings, it is possible to confirm that cultures in low adhesion conditions can be used to maintain stem characteristics for a longer time than traditional adhesion cultures. Immunophenotyping of MSCs derived from subcutaneous and visceral adipose tissue demonstrate that cell populations express well-defined MSC-associated surface markers CD29, CD73, CD90 and CD105 [20,21]. Western blot analysis confirmed the presence of stem cell markers; both ASCs were positive for SOX2, OCT3/4 and NANOG, which are the principal transcription factors that regulate pluripotency and plasticity. Our results indicate that the stemness of ASCs should be defined by their ability to differentiate into multiple lineages coupled with their expression of the pluripotent stem cell-related genes *OCT-4*, *SOX2* and *NANOG*. This datum could be considered useful to functionally distinguish ASCs as more stem cell-like [22]. We found that the embryonic stem cell marker *NANOG* is also over-expressed in MSCs derived from adipose tissue, confirming recent studies that have demonstrated a central role of *NANOG* in embryonic stem cells [23].

Stem cell pluripotency and differentiation are strictly controlled by a coordinated network of transcription factors [24]. Among them, *OCT4* and *NANOG* have been recognized as crucial transcriptional regulators of stem cell self-renewal during embryogenesis [25,26]. More recently, it has been shown that both *OCT4* and *NANOG* are also expressed by undifferentiated adult MSCs [27,28,29]. In ESCs, the over-expression of NANOG, the homeobox-containing transcription factor, is considered fundamental in maintaining the pluripotency and self-renewing characteristics of culture conditions inducing under-differentiation [24]. Recent studies have identified the downstream targets of *OCT4*, which include genes encoding for self-renewal factors, lineage-specific factors, signaling molecules and DNA damage response sensors [25]. Thus, *OCT4* is involved in a broad spectrum of cellular processes that collectively specify the self-renewal state of the ESCs. On the other side, the role of *SOX2* seems to be crucial, as well. Indeed, the lack of SOX2 leads ESCs to differentiation and to the loss of pluripotency property [30]. Based on these data, *NANOG* is considered a core element of the pluripotent transcriptional network. Transient downregulation of *NANOG* appears to predispose cells towards differentiation, but does not mark commitment. Therefore, unlike *OCT4* and *SOX2*, *NANOG* plays a pivotal role in the maintenance of the epiblast and ES cells by repressing differentiation along the primitive endoderm lineage. *SOX2* is capable of hetero-dimerizing with *OCT4* to mediate the transcription activities of several ES cell-specific genes including *NANOG* [31]. Interestingly, *OCT4* and *SOX2* are also involved in reciprocal regulation of each other’s expression [32]. OCT4 overexpression induces de-differentiation of ASCs into a more immature status by activating the AKT/phosphoinositide 3-kinase (PI3K) and extracellular signal-related kinase (ERK1/2) signaling pathways [33]. Indeed, it has been reported that ASCs possess their own multipotency to reprogram into more primitive stem cells [34]. However, how this process takes place mechanistically remains controversial. Suzuki et al showed that *NANOG* expression is not regulated by *Brachyury T* and *STAT3* in mouse ESCs [35]. In human ESCs, Vallier et al. showed that activin/nodal signaling stimulates the expression of *NANOG*, which in turn prevents FGF-induced neuroectoderm differentiation [36]. In addition, several studies have demonstrated that the OCT4/SOX2 complex is directly bound to the NANOG promoter for the target gene regulation [37]. Today, *OCT4*, *SOX2* and *NANOG* are known to bind the same regulatory regions in undifferentiated mouse and human ESCs, and these binding sites are often in close proximity to one another [38]. These studies suggest that OCT4, SOX2 and NANOG can physically interact with each other and coordinately regulate target genes [37,39].

In our experiments, we observed that downregulation of *NANOG* leads to significant downregulation of *OCT4* and *SOX2*, probably consistent with the loss of pluripotency. Indeed, in previous studies, downregulation of *NANOG* was shown to lead to significant downregulation of *OCT4* and loss of ES/EC cell-surface antigens; upregulation of several marker genes, including *GATA4*, *GATA6*, *LAMININ B1* and *AFP* [30,40,41] was reported. Moreover, the trophectoderm specification is indicated by upregulation of *CDX2*, *GATA2*, *hCG-α* and *hCG-β* [23,28,39,42,43].

All of these data confirm the use of adipose tissue as a potential source for multipotent cells and above all propose a suitable approach for future regenerative medicine and tissue engineering applications, as well as overall confirm its being a valuable resource in biotechnology.

## 4. Materials and Methods

### 4.1. Establishment of Adipose-Derived Stem Cell Cultures

Subcutaneous (SAT) and visceral (VAT) adipose tissue biopsies were obtained from 72 consenting patients (23 men, 47 women; age 45 ± 10; BMI range between 25 ± 0.5 and 35 ± 5), 42 obese and 28 non-obese subjects undergoing elective open-abdominal and laparoscopy surgery. The protocol was approved for RIMEDRI by the Independent Ethical Committee at the Azienda Ospedali Riuniti Villa Sofia-Cervello, No. 15, 11 December 2013, Palermo, Italy. All patients gave their written informed consent. Adipose tissue specimens were obtained from the subcutaneous and omental depots, and approximately 1 g of adipose tissue was taken from each fat depot. All biopsies were handled under sterile conditions and immediately used for subsequent preadipocyte isolation. Tissue specimens were immediately transported to the clean room of the laboratory of Regenerative Medicine (ISO 14644 Ec-GMP) in DMEM/Ham’s F12 1:1, dissected from fibrous material and visible blood vessels, cut into little fragments and incubated in PBS Ca^2++^/Mg^++^ (Phosphate-Buffered Saline with Calcium and Mangnesium) (Sigma Chemical, St. Louis, MO, USA) supplemented with 1 mg/mL collagenase type I (Sigma Chemical, St. Louis, MO, USA), with vigorous shaking (100 cycles/min) for 1 h at 37 °C. The resulting material was filtered through a 250-mm mesh, and adipocytes and free oil were separated from stromal vascular (SVF) components by centrifugation at 1200 rpm for 5 min at room temperature. The SVF pellet was resuspended in a growth medium consisting of DMEM/Ham’s F12 1:1 supplemented with 100 units/mL penicillin, 0.1 mg/mL streptomycin, 5% fetal calf serum (FCS), 1 ng/mL FGF-b and 10 ng/mL EGF.

### 4.2. Sphere Cultures

SVF cells were seeded at 1 × 10^2^ cell/cm^2^ in ultra-low adherent flasks (Corning, Avon, France) in defined culture medium, which consisted of DMEM/F12 supplemented with l-glutamine (2 mM), nonessential amino acids (1×), B27 (1×) (Invitrogen, Milan, Italy), human FGF-b (20 ng/mL), human EGF (20 ng/mL). Cells were incubated at 37 °C under 5% CO_2_, and half of the medium was changed once a week. Sphere formation was assessed by counting the number of spheres (cells > 3) under an optical microscope. To test if sphere-containing cells can revert to monolayer growth, spheres were dissociated with accutase (Sigma Chemical) and plated in flasks treated for cell culture (TPP) in ASC expansion medium.

### 4.3. Evaluation of Morphological Characteristics

Cells were fixed for 15 min at RT in 2% (*w*/*v*) paraformaldehyde, washed twice in distilled water and stained with crystal violet. Then, the cells were observed for their morphological features under a Zeiss phase contrast microscope (Zeiss, Gottingen, Germany) and photographed with a Nikon camera (Nikon, Firenze, Italy).

### 4.4. Flow Cytometry Analysis

Cells isolated from 12 samples of S-ASC and 12 samples of V-ASC were harvested and filtered through a 40-µm filter mesh and suspended at the concentration of 1 × 10^6^ cells/mL. Then, 100 µL of cell suspension containing 5 × 10^5^ cells were used for each flow cytometric test.

### 4.5. Immunophenotyping

Human anti-CD31, human anti-CD45, human anti-CD146, human anti-CD29, human anti-CD90 and human anti-CD105 (see Table 1) monoclonal antibodies were tested on S-ASC and V-ASC. The incubation conditions were in accordance with the manufacturer’s instructions. For anti-CD90 and anti-CD105, cells were washed twice with PBS/bovine serum albumin (BSA) 5% and incubated with Alexa Fluor 488 goat anti-mouse IgG (Invitrogen, Carlsbad, CA, USA) antibody for 1 h in the dark. Data were acquired on a FACS Calibur and analyzed using CELL Quest Pro software (Becton Dickinson, Franklin Lakes, NJ, USA).

### 4.6. Analysis of Cell Cycle Status of MSCs

Single-cell suspensions of 25 samples of S-ASC and 25 samples of V-ASC were obtained and seeded at a density of 2 × 10^3^ cells/cm^2^ (passage 3), and the DNA content was assessed according to Nicoletti’s protocol. Briefly, 1 × 10^6^ cells were fixed in 70% ethanol, rehydrated in PBS and then re-suspended in a DNA extraction buffer (with 0.2 M NaHPO_4_, 0.1% Tritonx-100 and, pH 7.8). Cells were stained with 1 μg/mL of propidium iodide (PI) for 5 min; fluorescence intensity was determined by analysis on a FACS Calibur (Becton Dickinson). Data were acquired with CellQuest Pro software (Becton Dickinson) software, and the percentages of G1, S and G2 phase cells were calculated with the MODFIT-LT 5.0 software program (Verity Software House Inc., Topsham, ME, USA).

### 4.7. RNA Isolation and Quantitative RT-PCR

mRNA from ASCs populations isolated from VAT and SAT biopsies derived from obese and normal weight patients were isolated using an RNeasy kit (Qiagen, Hamburg, Germany). Two hundred fifty nanograms of RNA from S-ASC and V-ASC were reverse-transcribed with standard reagents (Promega, Madison, WI, USA). One microliter of each reverse-transcription reaction was amplified using SYBR Green PCR master mix from Qiagen (Quantitect SYBR green master mix), using the RotorGene PCR system (Qiagen, Hamburg, Germany). For each gene, mRNA expression was normalized for the housekeeping gene β-actin. Amplification of specific transcripts was confirmed by melting curve profiles at the end of each PCR. PCR primers, *OCT4*, *NANOG*, *THY-1* (*CD-90*), *CD105* and *CD 73* were purchased from Qiagen (QuantiTect^®^ Primer Assays, Qiagen); primer for *SOX2* was purchased from MWG (Eurofins Genomics, Ebersberg, Germany); and primer for β-actin was purchased from Invitrogen (Table 2) All reactions were performed using the Quantitect SYBR Green PCR Kit (Qiagen) on the RotorGene Q Instrument (Qiagen). Each cDNA sample was mixed with specific primer sets and PCR master mix. The PCR reactions were performed using the following parameters for 40 cycles: denaturation at 95 °C for 3 min, 95 °C for 20 s, annealing at 60 °C for 30 s and elongation at 72 °C for 60 s. Reactions were performed at least in triplicate. The specificity of the amplified products was determined by melting peak analysis. The relative quantification model with efficiency correction was applied to normalize the expression of the target gene to β-actin (used as a housekeeping gene) and to compare gene expression with bone marrow mesenchymal stem cell(BM-MSCs) and with a commercial primary cell line immortalized with a human telomerase reverse transcriptase (hTERT) immortalized cell lines (ASC52telo, hTERT immortalized adipose-derived mesenchymal stem cells (ATCC^®^ SCRC-4000™, American Type Culture Collection, Manassas, VA, USA), used as a positive cell control. Relative expression levels for *ABCG2*, *OCT4*, *SOX2*, *CKIT*, *THY1*, *CD73*, *CD105* and *NANOG* were assessed using the 2^−ΔΔ*C*t^ method. The results were represented as histograms with GraphPad Prism 6 Software (GraphPad Software, Inc. La Jolla, CA, USA). The qRT-PCR analyses for the stem gene were also performed after siRNA transfection experiments.

### 4.8. siRNA Transfection

siRNAs transfection in 10 samples of S-ASC and in 10 sample of V-ASC was performed using the INTERFERin TM transfection agent (Polyplus-Transfection, Illkirch, France), according to the manufacturer’s instructions. Briefly, cells were seeded into six-well plates at a density of 250,000 cells/well or 96-well plates at a density of 3000 cells/well. The transfection agent and the siRNA complex were added to the cells and incubated for 72 h for expression analysis and 96 h for protein detection. The final concentration of SOX2 siRNA was 100 nM for mRNA analysis, 150 nM for protein detection and 40 nM for *NANOG* siRNA. Each assay was performed in triplicate in at least three independent experiments. *SOX2* was silenced using Stealth siRNA *SOX2* HSS144045 (Invitrogen). siCONTROL Stealth siRNA Negative Control was used as a control (Invitrogen). *NANOG* was silenced by *NANOG* siRNA (Santa Cruz Biotechnology, Dallas, TX, USA), and control siRNAs were used as a no-target control (Santa Cruz Biotechnology).

### 4.9. Western Blot Analysis

Proteins were extracted from adherent cultured cells isolated from 5 samples of S-ASC and 5 samples of V-ASC using RIPA buffer (50 mM Tris-HCl, pH 7.4, 150 mM NaCl, 1% Nonidet P40), supplemented with a protease inhibitor cocktail (Complete mini, Roche Diagnostics GmbH, Mannheim, Germany) and phosphatase inhibitors. Protein content was determined according to Bradford’s method. Proteins were separated by Bio-Rad (Bio-Rad, Segrate, Italy), electrotransferred to nitrocellulose membrane and blotted with the following primary antibodies: rabbit antihuman SOX2 (Poly6308, BioLegend, San Diego, CA, USA), mouse antihuman Oct-4 (sc-5279, Santa Cruz Biotechnology), goat antihuman NANOG (sc-30331, Santa Cruz Biotechnology), mouse anti-β-actin IgG1 (A5441, Sigma-Aldrich) Secondary antibodies were goat anti-rabbit IgG-HRP (sc-2030, Santa Cruz Biotechnology), goat anti-mouse IgG-HRP (sc-2031, Santa Cruz Biotechnology) and donkey anti-goat IgG-HRP (sc-2033, Santa Cruz Biotechnology) (see Table 3). Antigen-antibody complexes were visualized using the ECL prime (Amersham, GE Healthcare Europe GmbH, Milan, Italy) on a CCD camera (Chemidoc, Bio-Rad, Milan, Italy). Western blot bands were quantified with ImageJ 1.48 software (National Institutes of Health, Bethesda, MD, USA).

## 5. Conclusions

The use of adipose-derived stem cell is promising in regenerative medicine and tissue engineering applications and constitutes a valuable resource in biotechnology. Our aim was to study the different expression of embryonic stem cell markers *NANOG*, *SOX2* and *OCT3/4* in ASCs derived from SAT and VAT tissue and to explore whether *NANOG* possesses a more prominent role in this network. Our experiments confirm the presence of the principal transcription factors that regulate pluripotency and plasticity in both S-ASC and V-ASC cells. In spheres, *SOX2*, *OCT4* and *NANOG* genes were highly expressed when compared with adherent S-ASC and V-ASC cells. From our findings, NANOG seems to exert a hierarchical role in the network that regulates pluripotency, as its silencing causes downregulation of *OCT3/4* and *SOX2* genes in both S-ASCs and V-ASCs, while *SOX2* silencing does not affect *NANOG* and *OCT3/4* gene expression in both S-ASCs and V-ASCs. In the future, other methods will be necessary to further investigate whether *NANOG* downregulation in S-ASC and in V-ASC cells plays a role in the long-term differentiation of specialized cells. Finally, the lack of *OCT4* silencing represents a limit for the definition of the single role of each network component. In conclusion, our results outline the importance of these data for future applications of ASC cells in regenerative medicine.

## Figures and Tables

**Table 1:** Monoclonal antibodies used for the characterization of cell phenotypes.

Primary Antibody/Localization Marker	Code Number	Diluition	Incubation
CD31, surface	Miltenyi Biotec, 130-092-654	1:100	30 min, r.t.
CD45, surface	Miltenyi Biotec, 130-080-202	1:100	30 min, r.t.
CD146, surface	Miltenyi Biotec, 130-092-851	1:100	30 min, r.t.
CD90, surface	Chemicon, CBL415	1:50	o/n, r.t.
CD105, surface	Biolegend,323202	1:50	o/n, r.t.
CD29, surface	Miltenyi Biotec, 130-101-256	1:100	30 min, r.t.
Secondary antibody	Code Number	Diluition	Incubation
AlexaFluor 488	Life Technologies, Z25402	1:50	20 min, r.t.

Miltenyi Biotec, Bergisch Gladbach, Germany; Santa Cruz, Dallas, Texas; BioLegend, London, UK; Life Technologies, Carlsbad, CA, USA; o/n, overnight; r.t., room temperature.

**Table 2:** Real-time quantitative PCR primers used for gene expression investigation.

RNA	Primer Sequence	Code Number
*ABCG2*	Qiagen^®^	QT00073206
*NANOG*	Qiagen^®^	QT01844808
*OCT3/4*	Qiagen^®^	QT00210840
*SOX2*	FORWARD: 5′-GGAGACGGAGCTGAAGCCGC-3′ REVERSE: 5′-GACGCGGTCCGGGCTGTTTT-3′	
*THY1*	Qiagen^®^	QT00023569
*CD105*	Qiagen^®^	QT00013335
*CD73*	Qiagen^®^	QT00027279
β-Actin	FORWARD: 5′-GGACTT CGA GCA AGA GAT GG-3′ REVERSE: 5′-AGC ACT GTG TTG GCG TAC AG-3′	

**Table 3:** Antibodies used for characterization for stem cell markers.

**Primary Antibody/Localization Marker**	**Code Number**	**Dilution**	**Incubation**
NANOG, nuclear and cytoplasmatic	sc-30331, Santa Cruz Biotechnology	1:500	o/n, 4 °C
OCT3/4, nuclear	sc-5279 , Santa Cruz Biotechnology	1:500	o/n, 4 °C
SOX2 nuclear	Poly6308, BioLegend	1:500	o/n, 4 °C
**Secondary Antibody**	**Code Number**	**Diluition**	**Incubation**
Goat anti-rabbit IgG-HRP	sc-2030, Santa Cruz Biotechnology	1:2500	90 min, r.t.
Goat anti-mouse IgG-HRP	sc-2031, Santa Cruz Biotechnology	1:2500	90 min, r.t.
Donkey anti-goat IgG-HRP	sc-2033, Santa Cruz Biotechnology	1:2500	90 min, r.t.

Santa Cruz Biotechnology, Dallas, Texas; BioLegend, San Diego, CA; o/n, overnight; r.t., room temperature.

## Supplementary Materials

Supplementary materials can be found at www.mdpi.com/1422-0067/18/6/1107/s1.

## Abbreviations

S-ASC	Subcutaneous-adipose stem cell
V-ASC	Visceral-adipose stem cell
SVF	Stromal vascular fraction
