# Supplementary material for: NANOG Plays a Hierarchical Role in the Transcription Network Regulating the Pluripotency and Plasticity of Adipose Tissue-Derived Stem Cells
Source: Int J Mol Sci. 2017 May 23;18(6):1107. doi: 10.3390/ijms18061107 (PMC5485931; doi:10.3390/ijms18061107)
Supplement: Supplementary file 1 [file ijms-18-01107-s001.pdf]

Table S1. Calculated fold change values of mesenchymal stem cell genes in S-ASCs and V-ASCs in adhesion conditions. (** *p* <0.01)

**MSCs**	**MESENCHYMAL STEM CELL MARKERS**							
	ABCG2	OCT4	SOX 2	C-KIT	THY 1	CD-73	CD-105	NANOG
S-ASC	1.065 ± 0.1	0.9 ± 0.05	0.83 ± 0.07	17.13 ± 0.09	0.10 ± 0.03	0.64 ± 0.06	3.16 ± 0.9	2.7 ± 0.2**
V-ASC	1.11 ± 0.06	0.8 ± 0.05	0.93 ± 0.06	7.43 ± 0.07	0.42 ± 0.005	0.5 ± 0.003	2.38 ± 0.2	0.11± 0.8

Table S2. Stem cell transcription factor expression pattern in spheres derived from S-ASCs and V-ASCs in low adhesion vs. S-ASCs and V-ASCs in adhesion conditions.

**MSCs**	**MESENCHYMAL STEM CELL MARKERS**		
	NANOG	SOX 2	OCT4
S- ASC Sphere	2.84 ± 0.076	1.7 ± 0.36	3.08 ± 0.45
V-ASC Sphere	1.5 ± 0.196	0.86 ± 0.15	2.55 ± 0.47
S-ASC	1.92 ±0.28	0.53 ± 0.149	1.1 ± 0.26
V-ASC	0.4 ± 0.03	0.52 ± 0.13	0.32 ± 0.055

Table S3. Western blot analysis in the S-ASC and V-ASC cells.

**MSCs**	**MESENCHYMAL STEM CELL MARKERS**		
	NANOG	SOX 2	OCT4
S-ASCs	0.4 ± 0.01**	0.2 ± 0.02	0.163±0,01
V-ASCs	0.176 ± 0.001	0.158 ± 0.02	0.128 ± 0.01
